# A Case Study of Clinical Response to Rucaparib in a Patient with Metastatic Castration-Resistant Prostate Cancer and a *RAD51B Alteration*

**DOI:** 10.3390/curroncol29060333

**Published:** 2022-06-08

**Authors:** Brieuc Sautois, Andrea Loehr, Simon P. Watkins, Hélène Schroeder, Wassim Abida

**Affiliations:** 1 Medical Oncology, University Hospital of Liège, CHU Sart Tilman, 4000 Liège, Belgium; hschroeder@chu.ulg.ac.be; 2 Translational Medicine, Clovis Oncology Inc., Boulder, CO 08006, USA; aloehr@clovisoncology.com; 3 Clinical Science, Clovis Oncology UK, Ltd., Cambridge CB21 6GP, UK; swatkins@clovisoncology.com; 4 Genitourinary Oncology Service, Memorial Sloan Kettering Cancer Center, New York, NY 10065, USA; abidam@mskcc.org

**Keywords:** prostate cancer, PARP inhibitors, RAD51B

## Abstract

PARP inhibitors, such as rucaparib, have been well characterized in metastatic castration-resistant prostate cancer (mCRPC) associated with BRCA alterations, and the clinical activity of these agents has also been evaluated in patients with mCRPC associated with alterations in other non-BRCA DNA damage repair (DDR) genes, including *RAD51B*. There is likely a differential sensitivity to PARP inhibition based on the specific DDR gene altered, but research in this area is limited because of the low frequency of alterations in these genes. Here, we describe a mCRPC patient with a truncating rearrangement of *RAD51B* who had a radiographic and PSA response when treated with the PARP inhibitor rucaparib within the TRITON2 trial. We investigated the patients’ response parameters, circulating tumor DNA (ctDNA) fraction and tumor genomics longitudinally, using next-generation sequencing (NGS) of tissue and plasma. ctDNA fraction correlates with radiographic and PSA response and is lower during times of response. NGS did not reveal any potential genomic mechanism of acquired drug resistance. This case shows evidence for rucaparib activity in a rare patient with mCRPC and a *RAD51B* truncation.

## 1. Introduction

DNA repair is mediated by various proteins such as the poly(adenosine diphosphate [ADP]-ribose) polymerase (PARP) enzymes, which are crucial for single-strand DNA break repair, and by *BRCA1*, *BRCA2*, and *RAD51*, which are involved in homologous recombination repair (HRR) [1,2,3]. In tumor cells with impaired HRR (e.g., through gene alteration), the enzymatic inhibition of PARP proteins results in the accumulation of DNA damage and cell death through an interaction known as synthetic lethality [4,5,6].

Rucaparib is a PARP inhibitor approved in the United States for the treatment of patients with deleterious *BRCA1* or *BRCA2* (BRCA) mutation-associated metastatic castration-resistant prostate cancer (mCRPC) who have been treated with androgen receptor-directed therapy and a taxane-based chemotherapy [7]. The accelerated approval of rucaparib as a treatment for patients with mCRPC was based on the efficacy results from TRITON2 (NCT02952534), an international, multicenter phase II study of rucaparib in patients with mCRPC and homologous recombination repair deficiency (HRD) [8].

In addition to evaluating patients with a BRCA alteration, TRITON2 investigated rucaparib treatment in a smaller cohort of patients with mCRPC and with a non-BRCA DNA damage repair (DDR) gene alteration [9], including *RAD51B*, one of the *RAD51* paralogs involved in the HRR pathway [3,10]. Deleterious alterations of *RAD51B* in prostate cancer are rare and estimated to be present in roughly 0.56% of patients [11]. Here, we report a case study of a patient with mCRPC and a *RAD51B* rearrangement, which is of particular interest because data from patients with *RAD51B* alterations who were treated with a PARP inhibitor are limited due to the low prevalence.

## 2. Case Report

In April 2006, a 63-year-old man, a former smoker with no family history of cancer, was diagnosed with cT2 N0 M0 prostate cancer. The patient received a radical prostatectomy, and the pathology report classified the tumor as pT3a Nx adenocarcinoma with a Gleason score of 7 (3 + 4). 

**Figure 1:**
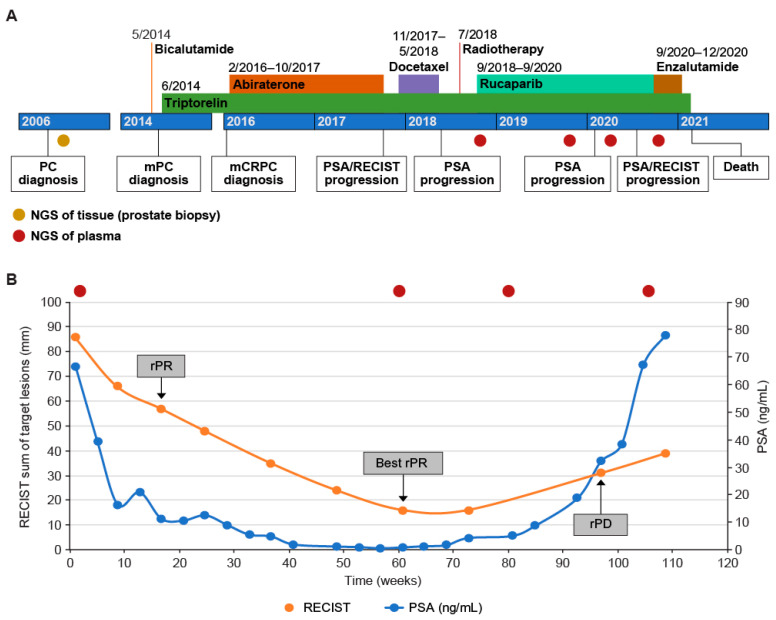
(**A**) The clinical course of the patient, and (**B**) the evolution of PSA and tumor measurements per RECIST with rucaparib. mCRPC, metastatic castration-resistant prostate cancer; mPC, metastatic prostate cancer; NGS, next-generation sequencing; PC, prostate cancer; PSA, prostate-specific antigen; RECIST, Response Evaluation Criteria In Solid Tumors, version 1.1; rPD, radiological progressive disease; rPR, radiological partial response.

**Figure 2:**
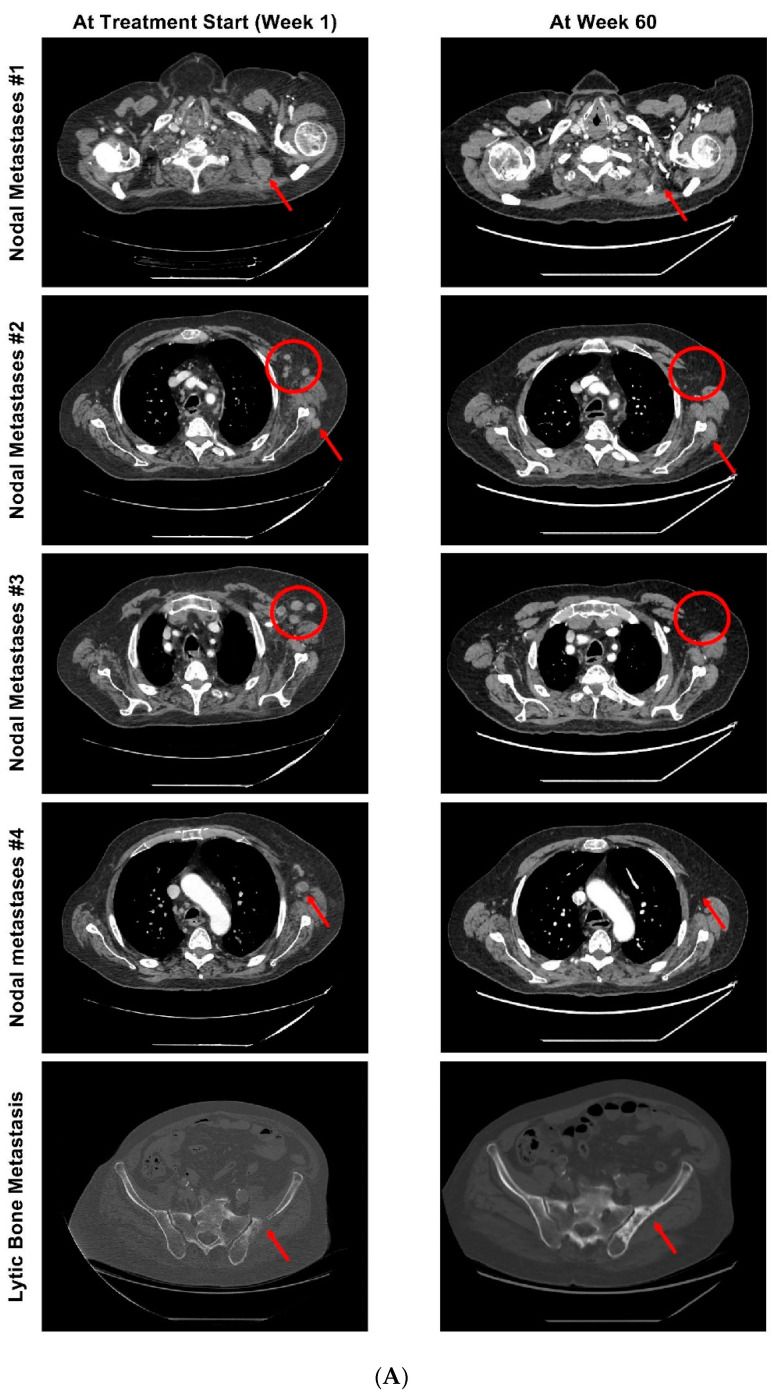

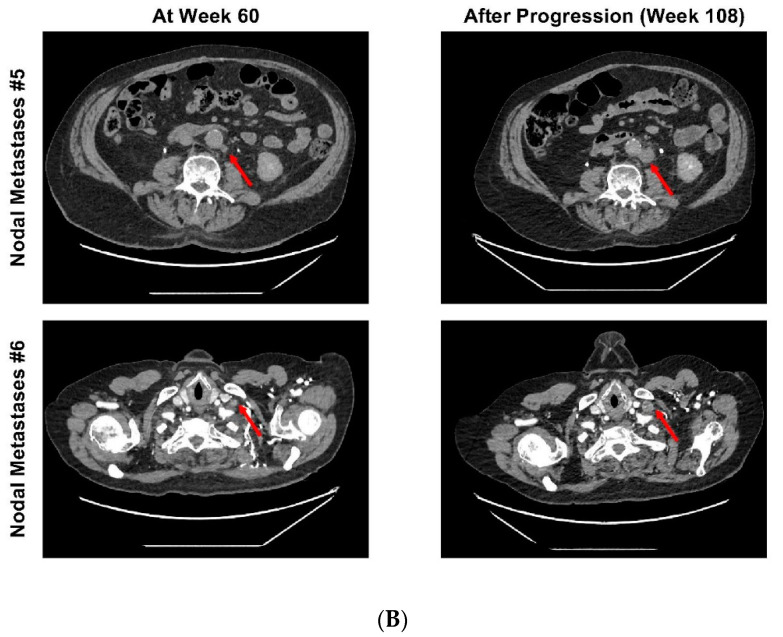
(**A**) Initial tumor scans (at start of rucaparib treatment) compared with scans at the time of best response (week 60 of rucaparib treatment) of nodal metastases and lytic bone metastases at the target lesions. (**B**) Scans at the time of best response (week 60 of rucaparib treatment) compared with scans after radiographic progression.

In May 2014, the patient was prescribed a short-course bicalutamide and started long-term triptorelin as an androgen deprivation therapy (ADT) for bone and lymph node metastases (Figure 1A). In February 2016, the confirmed diagnosis of mCRPC led to the addition of abiraterone to ADT. With this regimen, the patient had a best response of stable disease; however, the treatment was stopped in October 2017 (after 87 weeks of treatment) due to a rise in prostate-specific antigen (PSA) values (doubling time of 2 months), with confirmed progression in bone and lymph nodes. Docetaxel was initiated in November 2017, and the patient achieved a confirmed partial response per Response Evaluation Criteria In Solid Tumors, version 1.1 (RECIST), as well as a PSA decrease >50%. Treatment was discontinued in May 2018 (23 weeks, 8 cycles), and PSA values began to rise within 8 weeks, followed by pain that required palliative radiation (1 × 8 Gy) for a spinal cord compression in late July 2018. Nodal and bone disease progression was confirmed in August 2018 (Figure 2A).

In September 2018, the patient was enrolled in the TRITON2 study based on results from genomic testing with the FoundationOne next-generation sequencing (NGS) assay (Foundation Medicine, Cambridge, MA, USA) [12], which detected the presence of a truncating *RAD51B* rearrangement in an archival tumor tissue biopsy obtained at the time of diagnosis (June 2006; Table 1). The rearrangement was a fusion with *ACTN1* resulting in the deletion of exons 3 through 11 of *RAD51B*, which has a total of 11 exons. In addition, a pathogenic *TMPRSS2-ERG* fusion and a deleterious *RB1* rearrangement were detected. NGS of a plasma sample collected prior to rucaparib treatment using the FoundationOne Liquid CDx assay [13] detected the presence of additional somatic pathogenic alterations, among them 2 *TP53* mutations (Table 1, Table S1).

No other potential drivers of the disease were identified, and all detected alterations were confirmed to be of somatic origin using the Color Hereditary Cancer Test [14].

The patient started rucaparib at the recommended dose of 600 mg twice daily, but hematologic toxicity, particularly anemia, led to several treatment interruptions with subsequent dose reductions to 200 mg twice daily; the patient ultimately received rucaparib for 107 weeks (Figure 1A). At the start of TRITON2, the patient had more than 10 bone-associated lesions and multiple soft-tissue lesions in the left shoulder, left scapula, and left axillary and latero aortic lymph nodes. Treatment with rucaparib resulted in a confirmed partial response per modified RECIST and/or Prostate Cancer Clinical Trials Working Group 3 criteria (maximum of 81% decrease in target lesion diameters; Figure 1B and Figure 2A) lasting 80 weeks (December 2018 to July 2020), with no confirmed progression in the bone. The patient also had a confirmed PSA response (≥50% decrease from baseline confirmed by a second measurement ≥3 weeks later) (Figure 1B) lasting 64 weeks (October 2018 to January 2020) with a maximum decrease from the baseline of 99%. In comparison, 15 of 27 TRITON2 patients with a BRCA mutation and a radiographic response had a duration of response ≥6 months, and the median time to PSA progression was 28 weeks in all TRITON2 BRCA-mutated patients [8]. The patient discontinued due to clinical disease progression at new sites in the left subclavicular and para-aortic areas in September 2020 (Figure 2B). Following discontinuation of rucaparib treatment, the patient received enzalutamide 160 mg every day from September 2020 until December 2020 and died in February 2021.

The longitudinal genomic profile of the patient was assessed through genomic testing with the GuardantOMNI assay (Guardant Health, Redwood City, CA, USA) [15] of plasma samples collected at the start of treatment (September 2018 [week 1]), at the nadir of response (October 2019 [week 60]), after a rise in PSA following the confirmed response (March 2020 [week 80]), and following progression (September 2020 [week 108]) to further explore the genomic landscape. The *RAD51B* truncation was found in the archival tumor sample obtained at the time of initial diagnosis, in the plasma obtained prior to rucaparib treatment, and in the plasma after progression; however, the on-treatment plasma samples taken around the time of best response contained less than 2% of cell-free tumor DNA, and the *RAD51B* rearrangement was not detected due to the low (<10%) tumor fraction. The tumor fraction of all plasma samples was low and ranged from 1.3% at the time of the best radiographic response to 10.3% at the time of treatment (Table 1). In the progression sample, no secondary alterations of *RAD51B* or other apparent mechanisms of reversion were detected.

## 3. Discussion

While rucaparib and other PARP inhibitors have been perhaps best characterized in mCRPC associated with BRCA alterations, the clinical activity of these agents has been evaluated in patients with mCRPC associated with alterations in other non-BRCA DDR genes, including *RAD51B* [16]. In the phase III PROfound study (NCT02987543), while median progression-free survival was significantly longer with olaparib versus control agents in men with mCRPC who had alterations in BRCA or *ATM* (7.4 vs. 3.6 months; hazard ratio, 0.34 [95% CI, 0.25–0.47]; n = 245)*,* more modest effects were seen in the subgroup of patients with alterations in other DDR genes (4.8 vs. 3.3 months; hazard ratio, 0.88; n = 142) [16]. There is likely a differential sensitivity to PARP inhibition based on the specific DDR gene altered, but research in this area is hindered by the low frequency of these alterations: among the genomically selected PROfound patients, only 7 had an alteration in *RAD51B*, including one in the olaparib group with a co-occurring alteration in *ATM* and another in the control group with a co-occurring alteration in *BRCA2*. For patients who had a truncation *RAD51B* rearrangement without a co-occurring DDR gene alteration (n = 5), the median imaging-based progression-free survival was 10.9 months in the olaparib group (n = 4) and 1.8 months in the control group (n = 1) [16].

In the TRITON2 patient described here, we hypothesize the tumor response to rucaparib was likely driven by HRD caused by the truncating rearrangement of *RAD51B*. The *RAD51B* truncation was found in the archival tumor sample obtained at the time of initial diagnosis and in the plasma obtained prior to and post rucaparib treatment; however, the on-treatment plasma samples at time of response contained very little circulating tumor DNA, and the *RAD51B* rearrangement could not be detected. The tumor fraction of all plasma samples was low, and all plasma samples had reduced sensitivity for deletion calling. Therefore, the possibility of another undetected disease driver, such as homozygous BRCA loss, cannot definitively be ruled out.

Treatment with rucaparib resulted in radiographic and PSA responses, and following a dose reduction to mitigate adverse events, a continuation of treatment with rucaparib 200 mg twice daily allowed for the maintenance of the radiological response for over 1 year. The patient eventually developed new lesions in the left subclavicular and para-aortic areas. However, enlargement was not witnessed in the target lesions of the left axillary lymph nodes that were present at the start of rucaparib treatment, suggesting that the new lesions may have potentially been due to the emergence of new cancer clones. While the NGS analysis of postprogression plasma did not reveal any reversion mechanism or new genomic clones, in the absence of NGS data from these new lesions, it is not possible to confirm or reject this hypothesis.

In summary, this case shows evidence for rucaparib activity in the sole patient with a genomic alteration of *RAD51B* enrolled in TRITON2, adding further data to suggest that patients with mCRPC and alterations in DDR genes besides *BRCA1* or *BRCA2* may also experience clinical benefits from treatment with a PARP inhibitor.

## Figures and Tables

**Table 1:** Summary Of Longitudinal Genomic Testing.

Time Point	Sample Type	Assay	ctDNA Fraction	*RAD51B/ACTN1* Rearrangement	*TMPRSS2-ERG* Fusion	*TP53* Y220C
Archival	Tissue	FoundationOne	NA	Detected	Detected	Not detected
Pre-treatment	Plasma	FoundationOne Liquid	15.5%	Detected	Detected	Detected
Pre-treatment	Plasma	Guardant Omni	10.3%	Detected	Detected	Detected
On-treatment Week 60	Plasma	Guardant Omni	1.3%	Not Detected	Not Detected	Detected
On-treatment Week 80	Plasma	Guardant Omni	2.1%	Not Detected	Not Detected	Detected
Post-progression Week 106	Plasma	Guardant Omni	9.6%	Detected	Detected	Detected

Abbreviations: AF, allelic fraction; ctDNA, circulating tumor DNA.

## Data Availability

Requests for de-identified datasets for the results reported in this publication will be made available to qualified researchers following submission of a methodologically sound proposal to medinfo@clovisoncology.com. Data will be made available for such requests following online publication of this article and for 1 year thereafter in compliance with applicable privacy laws, data protection, and requirements for consent and anonymization. Data will be provided by Clovis Oncology. Clovis Oncology does not share identified participant data or a data dictionary.

## Supplementary Materials

The following supporting information can be downloaded at: https://www.mdpi.com/article/10.3390/curroncol29060333/s1. Table S1. Supplemental Data.
